# Investigating the Causality and Pathogenesis of Primary Sclerosing Cholangitis in Colorectal Cancer Through Mendelian Randomization and Bioinformatics

**DOI:** 10.1155/genr/5887056

**Published:** 2025-05-18

**Authors:** Jie Jiao, Honglei Wang, Danping Sun, Wenbin Yu

**Affiliations:** Department of General Surgery, Qilu Hospital of Shandong University, Jinan 250012, Shandong, China

**Keywords:** bioinformatics analysis, colorectal cancer, Mendelian randomization, primary sclerosing cholangitis

## Abstract

**Introduction:** The relationship between autoimmune diseases and cancer risk has been increasingly studied. Colorectal cancer, a common malignancy with high morbidity and mortality, has been closely linked to inflammatory bowel disease (IBD) in previous research. However, the association and pathogenesis between primary sclerosing cholangitis (PSC) in autoimmune diseases and colorectal cancer remain incompletely understood. Our study directly investigated the relationship between PSC and colorectal cancer, excluding the influence of IBD, and provided new insights into this association.

**Methods:** Mendelian randomization (MR) analysis was first used to investigate the potential causal relationship between PSC and colorectal cancer. Sensitivity analyses were performed to verify the reliability of the MR results. Transcriptomic data were then analyzed based on the Cancer Genome Atlas (TCGA) and the Gene Expression Omnibus (GEO) database, combined with clinical prognostic data for the final identification of core differential genes.

**Results:** MR analysis demonstrated that genetic susceptibility to PSC was associated with an increased risk of colorectal cancer in a European population cohort (ratio: 1.038, 95% confidence interval: 1.016–1.060, and *p* < 0.001). Furthermore, sensitivity analyses confirmed the robustness of the MR results. Univariate and multivariate Cox analyses identified five core genes: NEDD4L, PPP1R1A, NRG1, KCNJ16, and NECAB2. Patients grouped according to high or low expression of NRG1 showed significant differences in their prognosis (*p* < 0.001).

**Conclusion:** Our MR study provides evidence that genetic susceptibility to PSC is significantly associated with an increased risk of colorectal cancer in European populations. Analysis of transcriptomic data suggests that NRG1 can be used as a novel biomarker to predict patient prognosis when colorectal cancer and PSC coexist.

## 1. Introduction

In recent years, the relationship between cancer and the immune system has emerged as a significant research focus. On one hand, inflammation contributes to tumorigenesis; on the other hand, activation of the immune system possesses robust antitumor capabilities [1–3]. Primary sclerosing cholangitis (PSC) is a chronic, progressive cholestatic liver disease characterized by inflammation, fibrosis, and narrowing of the bile ducts, leading to progressive liver dysfunction [4]. The etiology of PSC remains unknown, and it is classified as an autoimmune disease (AID) [5]. T-cell infiltration, a hallmark of PSC [6, 7], is closely associated with inflammatory bowel disease (IBD) [8], which also elevates the risk of colorectal cancer [9–11].

Colorectal cancer is a malignant disease characterized by high incidence and mortality rates [12, 13]. As the population ages, the prevalence of colorectal cancer continues to increase [14]. The etiology of colorectal cancer is multifactorial, encompassing genetic predispositions, obesity, dietary habits, and intestinal flora [15, 16]. The immune system plays a pivotal role in the development, progression, and treatment of colorectal cancer. However, the link between PSC and colorectal cancer remains underexplored, necessitating further investigation through more rigorously designed studies.

Mendelian randomization (MR) is an epidemiological method that accurately assesses causality between diseases [17, 18]. Unlike randomized controlled trials, MR effectively addresses potential confounders and reverses causality that can introduce bias [19]. This method employs single nucleotide polymorphisms (SNPs) as instrumental variables (IVs) to establish causality, independent of environmental risk factors, and determines relationships before disease progression, thereby mitigating reverse causality concerns [20–22]. In addition, the rapid advancement of high-throughput technologies offers a unique opportunity to explore the underlying mechanisms of PSC and colorectal cancer in depth.

In this study, we utilized genome-wide association study (GWAS) statistics and transcriptomics data from previous research to examine the potential causal relationship between PSC and colorectal cancer through MR analysis. In addition, we employed weighted gene coexpression network analysis (WGCNA) to identify common differentially expressed genes (DEGs) between the two conditions, aiming to elucidate the genetic mechanisms involved.

## 2. Methods

### 2.1. Study Design

**Figure 1:**
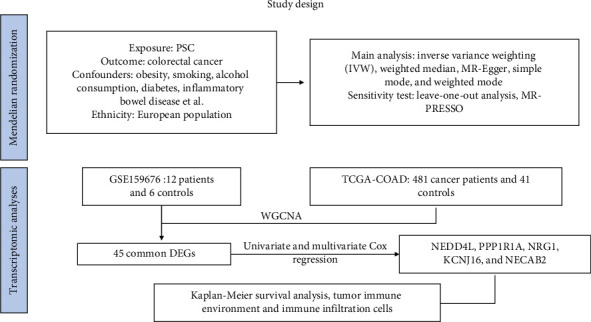
Overview of study design.

The causal relationship between the exposure factor (PSC) and the outcome (colorectal cancer) was established using two-sample MR. Subsequently, molecular mechanisms were explored by examining DEGs shared between the two diseases. The study's conceptual framework is illustrated in Figure 1.

### 2.2. Data Sources

For MR, we acquired data from GWAS meta-analyses of European populations diagnosed with PSC, consisting of 14,890 subjects (ieu-a-1112) [23]. For colorectal cancer, we selected data from a recent European population-based GWAS, involving 69,175 participants (GCST90129505) [24].

For transcriptomic analyses, microarray expression and clinical data for PSC were retrieved from the Gene Expression Omnibus (GEO) database (accession number GSE159676), comprising data from 12 patients and 6 controls. RNA sequencing data and clinical information for colorectal cancer were sourced from the Cancer Genome Atlas (TCGA) database (Project: TCGA-COAD), encompassing 481 cancer patients and 41 controls.

### 2.3. MR

For the MR analysis, three critical assumptions must be satisfied [25]: (1) relevance: the IVs are closely related to PSC; (2) independence: the IVs are not associated with any confounders in the PSC-colorectal cancer relationship; and (3) exclusivity: the IVs influence the colorectal cancer solely through the PSC, not via alternative pathways.

SNPs strongly associated with PSC were collected from selected GWAS data requiring a *p* value less than 5 × 10^−8^. To ensure the independence of SNPs, linkage disequilibrium (LD) screening was conducted with an *r*^2^ threshold of less than 0.001 and a clumping distance greater than 10,000 kb. IVs exhibiting weak instrument bias were subsequently excluded if their F-statistics exceeded 10. To mitigate the impact of confounding factors, exclusions were performed using data from PhenoScanner, focusing on factors such as obesity, smoking, alcohol consumption, diabetes, IBD, and cholesterol levels [26–28]. The remaining SNPs were then utilized as IVs for subsequent analyses.

We investigated the causal association between PSC and colorectal cancer using five prevalent MR approaches: inverse variance weighting (IVW), weighted median, MR-Egger, simple mode, and weighted mode [29–31]. IVW, noted for its high statistical efficacy, was considered the most reliable for causal estimation and thus served as the primary method of assessment. The remaining four methods were employed as complementary tools to address potential biases [29, 32]. In addition, sensitivity analyses were performed to confirm adherence to the hypothesized conditions, including assessing heterogeneity with Cochran's Q-test [33]. The MR-PRESSO method was employed to identify and correct for outliers and horizontal pleiotropy in IVs [34]. The MR-Egger intercept test was utilized to detect horizontal and directional pleiotropy in IVs. Furthermore, leave-one-out analyses were conducted to evaluate the impact of individual SNPs on causality or bias.

### 2.4. Transcriptomic Analyses

#### 2.4.1. Identification of DEGs

To identify DEGs within the PSC dataset GSE159676, we utilized the “limma” package [35], filtering for genes with |log_2_FoldChange| greater than 1 and adjusted *p* values less than 0.05. This approach identified 245 DEGs, comprising 145 upregulated and 100 downregulated genes. Similarly, in the TCGA dataset, genes were screened using identical criteria, followed by employing WGCNA to construct networks [36]. Two significant coexpression networks were identified based on their correlation with clinical characteristics, incorporating a total of 3878 DEGs. The intersection of these DEGs, consisting of 45 genes, was selected for further analysis.

#### 2.4.2. Functional Enrichment Analysis and Protein–Protein Interaction (PPI)

To elucidate the biological mechanisms of DEGs, gene ontology (GO) analysis and network analysis of genes and pathways were conducted using the “ClusterProfiler” package [37]. In addition, the PPI network was analyzed using the STRING database (https://string-db.org).

#### 2.4.3. Association With Prognosis

The minimum absolute shrinkage and selection operator (LASSO) regression [38], implemented using the “glmnet” package, was employed to identify DEGs associated with overall survival (OS) in colorectal cancer, resulting in a subset of seven prognostically relevant genes. Subsequent univariate and multivariate Cox regression analyses identified five genes—NEDD4L, PPP1R1A, NRG1, KCNJ16, and NECAB2—with strong prognostic associations, all demonstrating *p* values less than 0.05. Patients were categorized into high and low expression groups based on median gene expression levels, and OS differences were analyzed using Kaplan–Meier curves. Prognostic accuracy was evaluated through time-dependent receiver operating characteristic (ROC) analysis.

#### 2.4.4. Tumor Immune Environment and Immune Infiltration Cells

The CIBERSORT method was employed to quantify the infiltration of 22 immune cell types within the tumor immune microenvironment of each sample [39]. Samples were categorized based on the median expression of each DEG, and comparisons were made to assess variations in immune cell distribution between the two groups.

### 2.5. Statistical Analysis

All data analysis in this study was conducted using R software (Version 4.3.1). MR analysis was carried out using the “TwoSampleMR” and “MR-PRESSO” packages. Bioinformatics analysis was performed utilizing the appropriate packages for each algorithm. Multiple comparisons were adjusted for false discovery rate (FDR) corrections (*p* < 0.05) [40], deemed evidence of statistical significance. For nonmultiple comparisons, two-sided *p* values less than 0.05 were considered statistically significant.

## 3. Results

### 3.1. Instrumental Variables Selection

For PSC, 11 associated SNPs were identified for MR analysis after excluding SNPs with echo structure effects and confounding factors. All SNPs demonstrated an F-statistic greater than 10. Following significant findings of heterogeneity and horizontal pleiotropy in Cochran's Q-test and the MR-PRESSO global test, two outliers, rs41316239 and rs72837826, were excluded. Subsequent SNP analyses indicated no heterogeneity (*p*=0.408) and absence of pleiotropy (*p*=0.986). Consequently, these 9 SNPs were utilized as the final IVs (Table S1).

### 3.2. Causality and Sensitivity Analyses

**Figure 2:**
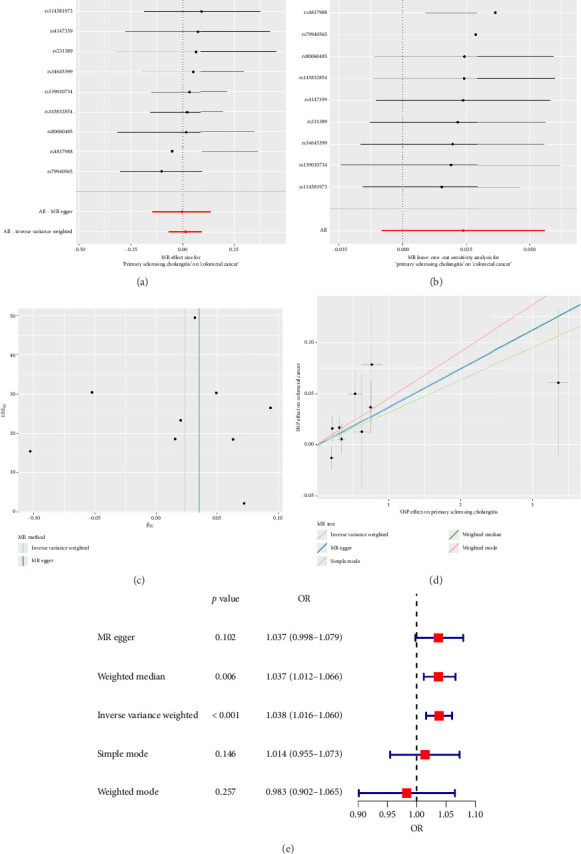
Causal relationship between PSC and colorectal cancer risk in European populations. (a) Forest plot: red dots represent the combined estimates of all SNPs using the IVW method, with horizontal lines depicting the 95% confidence interval. (b) Leave-one-out analysis: black dots show causal effects assessed using the IVW method, excluding individual specific variants from the analysis, while red dots indicate IVW estimates using all SNPs. (c) Funnel plot: vertical lines represent estimates for all SNPs, and the symmetry of the funnel plot indicates no significant horizontal pleiotropy. (d) Scatterplot: the slope of each line represents the estimated effect of each MR randomization method. (e) Forest plot of ORs for the 5 MR methods.

The forest plot of SNPs (Figure 2(a)) indicated that the combined estimates of SNPs were collectively analyzed. The absence of sensitivity analyses in Figure 2(b) confirmed that no IVs influenced the overall outcomes of the MR analyses. The funnel plot (Figure 2(c)) exhibited symmetry, indicating no significant horizontal pleiotropy. Scatter plots (Figure 2(d)) illustrated the causal relationship between PSC and colorectal cancer using various MR methods. Employing the IVW method revealed that PSC significantly increases the risk of CRC, establishing a significant causal connection (IVW OR = 1.038, 95% CI = 1.016–1.060, and *p* < 0.001). The MR-Egger method indicated a similar trend in causality; however, it did not reach the threshold for statistical significance.

### 3.3. Identification of Common DEGs

**Figure 3:**
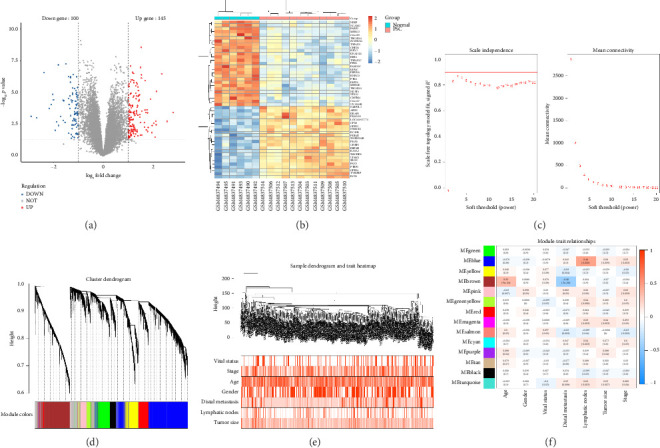
(a) Volcano plot illustrating the expression patterns of DEGs in the GSE159676 dataset, with blue indicating downregulated genes and red indicating upregulated genes. (b) Heatmap displaying the top 50 differential genes in the GSE159676 dataset. (c) Scale-free exponential analysis with varying soft threshold powers, identifying 3 as the appropriate soft threshold power in TCGA. (d) Dendrogram of genes clustered by the heterogeneity measure, grouping genes into 15 modules with different colors. (e) Dendrogram of samples postclustering. (f) Plot showing correlations between the 14 color modules and clinical features, with *p* values in parentheses (gray modules not shown).

**Figure 4:**
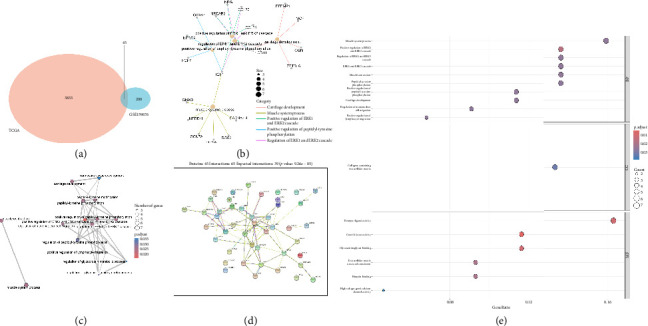
(a) Venn diagram showing the 45 differential genes common to both datasets. (b) Network diagram depicting gene interactions in the GO enrichment analysis. (c) Network diagram illustrating pathway interactions in the GO enrichment analysis. (d) PPI network diagram of the 45 differential genes. (e) Gene ontology enrichment analysis results.

After normalizing the RNA data, the GSE159676 dataset identified 245 DEGs (Figure 3(a)). Further analysis of the TCGA data using WGCNA with a soft-threshold efficacy of 3 revealed two significant modules (brown and blue) comprising a total of 3878 DEGs (Figures 3(b), 3(c), 3(d), 3(e), and 3(f)). Collectively, these modules encompass 45 key DEGs of interest (Figure 4(a)).

### 3.4. Enrichment Analyses and PPI

To elucidate the potential biological functions of key genes, we conducted GO enrichment analysis and network analysis of gene nodes and pathways (Figures 4(b) and 4(c)). The PPI network analysis is depicted in Figure 4(d). GO analysis showed DEGs were enriched in muscle system process, collagen-containing extracellular matrix, and receptor ligand activity (Figure 4(e)).

### 3.5. Survival Analysis

**Figure 5:**
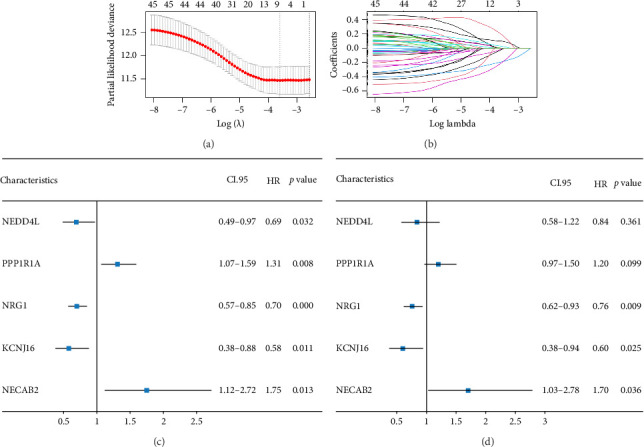
(a-b) Least absolute shrinkage and selection operator (LASSO) regression models prevent overfitting of recurrent features and identify key DEGs. (c-d) Results of univariate and multivariate COX analyses, displaying only genes with *p* < 0.05 in the univariate COX analysis.

**Figure 6:**
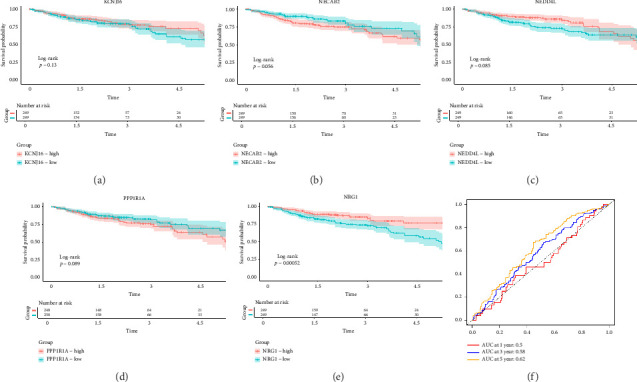
(a–e) K–M survival prognostic curves for the five genes KCNJ16, NECAB2, NEDD4L, PPP1R1A, and NRG1 are illustrated. (f) The ROC curve for NRG1 is displayed.

The 45 hub genes were refined using LASSO regression. Figures 5(a) and 5(b) illustrated comprehensive details on the selected LASSO model parameters and coefficient characteristics. Subsequently, nine genes advanced to univariate Cox analysis, among which five exhibited *p* values < 0.05 (Figure 5(c))—NEDD4L, PPP1R1A, NRG1, KCNJ16, and NECAB2. The outcomes of the multivariate Cox analysis indicated that NRG1, KCNJ16, and NECAB2 exhibited statistically differences (*p* < 0.05). The 5 genes were categorized into two groups—high and low expression—based on their median expression levels. The impact on overall survival between these groups was illustrated in Figures 6(a), 6(b), 6(c), 6(d), and 6(e), with the NRG1 grouping showing a significant association (*p* < 0.01) where higher expression correlated with improved survival. Corresponding ROC curves were also presented in Figure 6(f).

### 3.6. Immune Cell Infiltration Analysis

**Figure 7:**
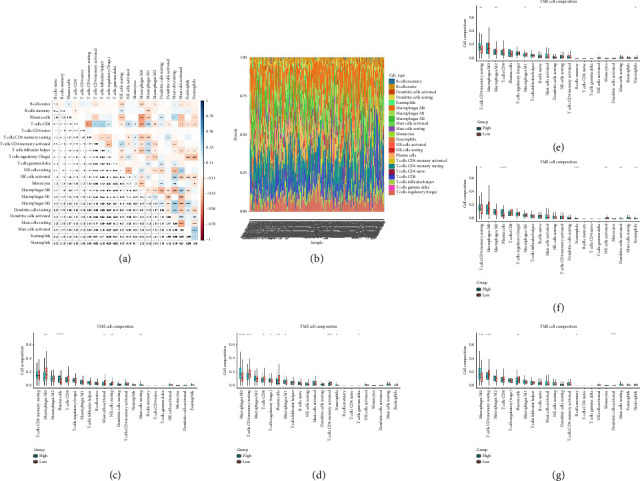
(a) Correlation diagram of infiltration for 22 immune cells. (b) Histogram showing the proportion of immune cells across all samples. (c–g) Box plots comparing differences in immune cell infiltration between high and low expression groups based on the median expression of the five core DEGs (NEDD4L, PPP1R1A, NRG1, KCNJ16, and NECAB2). ^∗^Indicates *p* < 0.05, ^∗∗^indicates *p* < 0.01, ^∗∗∗^indicates *p* < 0.001, and ^∗∗∗∗^indicates *p* < 0.0001.

Figure 7(a) illustrated the correlation among 22 different immune cells in colorectal cancer patients from TCGA. Figure 7(b) depicted the proportion of various immune cells across all patients. Figures 7(c), 7(d), 7(e), 7(f), and 7(g) demonstrated the differences in immune cell profiles following the stratification of patients based on the expression of 5 hub genes (NEDD4L, PPP1R1A, NRG1, KCNJ16, and NECAB2). Notably, in the NRG1 high expression group, the levels of CD4 memory resting T cells and naive B cells were significantly elevated whereas both M2 and M1 macrophages showed significant reductions.

## 4. Discussion

Alterations in the immune system play a critical role in the progression of cancer. PSC is a complex immune-related liver disease with an incompletely understood pathogenesis. Immune dysfunction, particularly T-cell dysfunction, is a key factor in the development of PSC, though the specific regulatory mechanisms remain unclear. Given the relatively low incidence of PSC, robust evidence linking it to tumorigenesis mechanisms is scarce. To mitigate potential confounders and reverse causality, our MR analysis was conducted to determine the causal relationship between PSC and the risk of colorectal cancer.

Our results aligned with findings from a prior observational study that identified an association between PSC and an increased susceptibility to colorectal cancer. The results of Aiva's study [41], which included over 1000 patients and 10,000 controls, demonstrated that patients with PSC had a four-fold increased risk of developing cancer overall and a seven-fold increased risk of developing colorectal cancer (HR: 7.5 and 95% CI: 5.6–10.0). It is noteworthy that nearly 90% of the PSC patients in this study were also diagnosed with IBD. Prior study suggested that patients with both PSC and IBD have a higher likelihood of developing colorectal cancer than those with IBD alone [42, 43]. IBD is a known risk factor for colorectal cancer, and PSC may either directly increase the risk of colorectal cancer or indirectly promote colorectal cancer through its association with IBD [44]. However, the precise nature of this relationship remains unclear. Our study directly excludes the effect of IBD and confirms that PSC independently contributes to the increased risk of colorectal cancer, thereby making the results more robust.

We hypothesized that PSC may contribute to colorectal cancer through mechanisms related to impaired hepatic bile acid excretion, common in PSC and other cholestatic disorders [45]. This impairment may lead to colonic accumulation of bile acids [46]. Secondary bile acids, known to cause DNA damage and promote cellular mutations, have been linked to a higher prevalence of colorectal cancer, particularly in the right proximal colon where their concentrations are highest [47, 48]. Treatment with ursodeoxycholic acid reduces levels of the secondary bile acid, decreases susceptibility to colitis, and exerts a therapeutic effect on cancer, thereby counteracting the carcinogenic effects of bile acids [49]. Another possible explanation is that disturbances in the intestinal flora of PSC patients increase the risk of colorectal cancer by disrupting the intestinal barrier and causing bacterial translocation. *Klebsiella pneumoniae*, known to damage the intestinal epithelium, is prevalent in the intestines of PSC patients, along with other abnormal bacilli and cocci [50]. In addition, changes in the composition of intestinal fungi further support the role of dysbiosis in promoting colorectal cancer in PSC [51].

Considering the detrimental effects of PSC on colorectal cancer, we conducted WGCNA using RNA data from two databases, identifying 45 DEGs. These genes were associated with pathways including muscle system processes, collagen-containing extracellular matrix, and receptor ligand activity. These findings align with earlier studies suggesting that abnormalities in the muscle system increase the likelihood of malignant disease in colorectal cancer patients [52]. Furthermore, ligand-receptor interactions, which are crucial for intercellular communication, play a significant role in the development and treatment of colorectal cancer [53].

Based on clinical prognostic data from TCGA, we identified five key targets: NEDD4L, PPP1R1A, NRG1, KCNJ16, and NECAB2. Several studies have demonstrated the role of these genes in colorectal cancer. NEDD4L downregulates and inhibits classical Wnt signaling in colorectal cancer [54]. PPP1R1A, an inhibitor of protein phosphatase-1, is associated with oncogenic effects and is commonly implicated in the progression and metastasis of Ewing's sarcoma [55]. However, evidence of PPP1R1A's impact on colorectal cancer remains elusive and requires further investigation. Mesenchymal stem cells stimulated colorectal cancer invasion, survival, and tumorigenesis by releasing soluble NRG1 [56]. NECAB2, primarily involved in regulating neuronal calcium homeostasis [57], and KCNJ16, which controls potassium ion flow [58], have not been previously linked to colorectal cancer development. We grouped these five genes according to their expression levels and found that the tumor microenvironment differed between the two groups. The samples were obtained from the tumor tissues of colorectal cancer patients. Notably, the infiltration of T cells CD4 memory resting cells was insignificant only in the NEDD4L grouping, while it was significant in the other four gene groupings. Given that a prominent feature of PSC is the abnormality of T cell infiltration, this link requires further investigation.

Our study has several limitations. First, despite employing various MR methods, we could not entirely eliminate bias due to potential horizontal pleiotropy. However, the direction and magnitude of MR estimates from methods such as IVW, weighted median, and MR-Egger were consistent across this research. Second, while we established a genetic association between PSC and an increased risk of colorectal cancer in European populations and identified DEGs, the underlying mechanisms remain elusive and require further investigation through more comprehensive basic research. In addition, it is essential to verify whether these results are consistent across different ethnic populations. Finally, the possibility of a nonlinear association between PSC and colorectal cancer cannot be ruled out and warrants confirmation through animal experiments or extensive cohort studies.

## 5. Conclusion

Our MR analyses indicated that patients with PSC were more likely to develop colorectal cancer in European populations. In addition, this study suggests that the NRG1 gene may play a significant role in the co-occurrence of these diseases, offering insights for potential targeted therapies.

## Data Availability

All data were sourced from freely accessible public databases, available for download at the following URL: https://www.ebi.ac.uk/gwas/studies/GCST90129505; https://gwas.mrcieu.ac.uk/datasets/ieu-a-1112/; https://portal.gdc.cancer.gov/; https://www.ncbi.nlm.nih.gov/geo/query/acc.cgi?acc=GSE159676.

## Supporting Information

Table S1 shows the specific parameters of the 9 SNPs used as IVs.
