# Supplementary material for: Investigating the Causality and Pathogenesis of Primary Sclerosing Cholangitis in Colorectal Cancer Through Mendelian Randomization and Bioinformatics
Source: Genet Res (Camb). 2025 May 18;2025:5887056. doi: 10.1155/genr/5887056 (PMC12103968; doi:10.1155/genr/5887056)
Supplement: Supporting Information — Additional supporting information can be found online in the Supporting Information section. [file 5887056.f1.docx]

Table S1. 9 SNPs used as instrumental variables for PSC in MR analysis

| SNP | A1 | A2 | β | SE | P-value | F-statistics |
| --- | --- | --- | --- | --- | --- | --- |
| rs114581973 | C | T | 0.5342 | 0.1020 | 3.40E-08 | 27.42 |
| rs139010734 | T | C | 3.3554 | 0.1400 | 1.98E-154 | 574.41 |
| rs145832854 | G | A | 0.6243 | 0.1190 | 2.58E-08 | 27.53 |
| rs231389 | C | T | 0.2062 | 0.0360 | 4.42E-09 | 32.81 |
| rs34645399 | G | A | 0.7467 | 0.0470 | 1.64E-59 | 252.40 |
| rs4147359 | A | G | 0.2167 | 0.0300 | 4.06E-13 | 52.19 |
| rs4817988 | G | A | 0.3148 | 0.0410 | 4.20E-15 | 58.96 |
| rs79940565 | C | T | 0.7631 | 0.1460 | 2.00E-08 | 27.32 |
| rs80060485 | C | T | 0.3457 | 0.0620 | 8.54E-09 | 31.09 |
